# Comparative genomics of *Rhodospirillum rubrum* S1 lineages reveals significant differences from the reference genome

**DOI:** 10.17912/micropub.biology.002395

**Published:** 2026-10-20

**Authors:** Sebastian Pöhl, Anke Treuner-Lange, Martin Thanbichler

**Affiliations:** 1 Department of Biology, Marburg University, Marburg, Germany; 2 Max Planck Institute for Terrestrial Microbiology, Marburg, Germany; 3 Microbes-for-Climate (M4C) Cluster of Excellence & SYNMIKRO, Marburg, Germany

## Abstract

Laboratory cultivation imposes selective pressures that drive rapid bacterial evolution, compromising experimental reproducibility. Here, comparative phenotypic and genomic analyses revealed significant divergence between two *Rhodospirillum rubrum* S1 lineages originally obtained from the same culture collection. Although both shared baseline variations relative to the reference genome, their trajectories diverged. A recently sourced line (DSM 467) exhibited few additional mutations, including a *fliF* frameshift abolishing motility. Conversely, a long-maintained laboratory line remained motile but harbored numerous unique variants, particularly within stress-responsive histidine kinases. These results demonstrate passaging-induced genomic drift, highlighting the necessity of cryopreservation and minimal serial cultivation to ensure strain fidelity.

**Figure 1. Comparison of R. rubrum S1 laboratory strains:** **(a)** Transmission electron micrographs showing representative cells of the *R. rubrum* S1 MT and DS strains, alongside a derivative of the MT strain (SP343), in which the frameshift mutation in *fliF* was corrected to restore the wild-type *fliF* allele matching the *R. rubrum* S1 reference genome sequence. Bar: 1 µm. Arrowheads indicate attached and sheared flagella. **(b)** Sche­matic mapping of unique and shared genomic deviations in the MT and DS laboratory strains relative to the *R. rubrum* S1 refer­ence genome (ATCC 11170). The position of the frameshift mutation in *fliF* is indicated. Due to the close proximity of certain mutations, individual bars may indicate multiple closely spaced genomic variants. **(c)** Summary of unique and shared genomic variants in the two labor­atory strains. Mixed variants are defined as loci where more than 25% of the reads support the reference genome sequence. Details are provided in the **Extended Data** file.

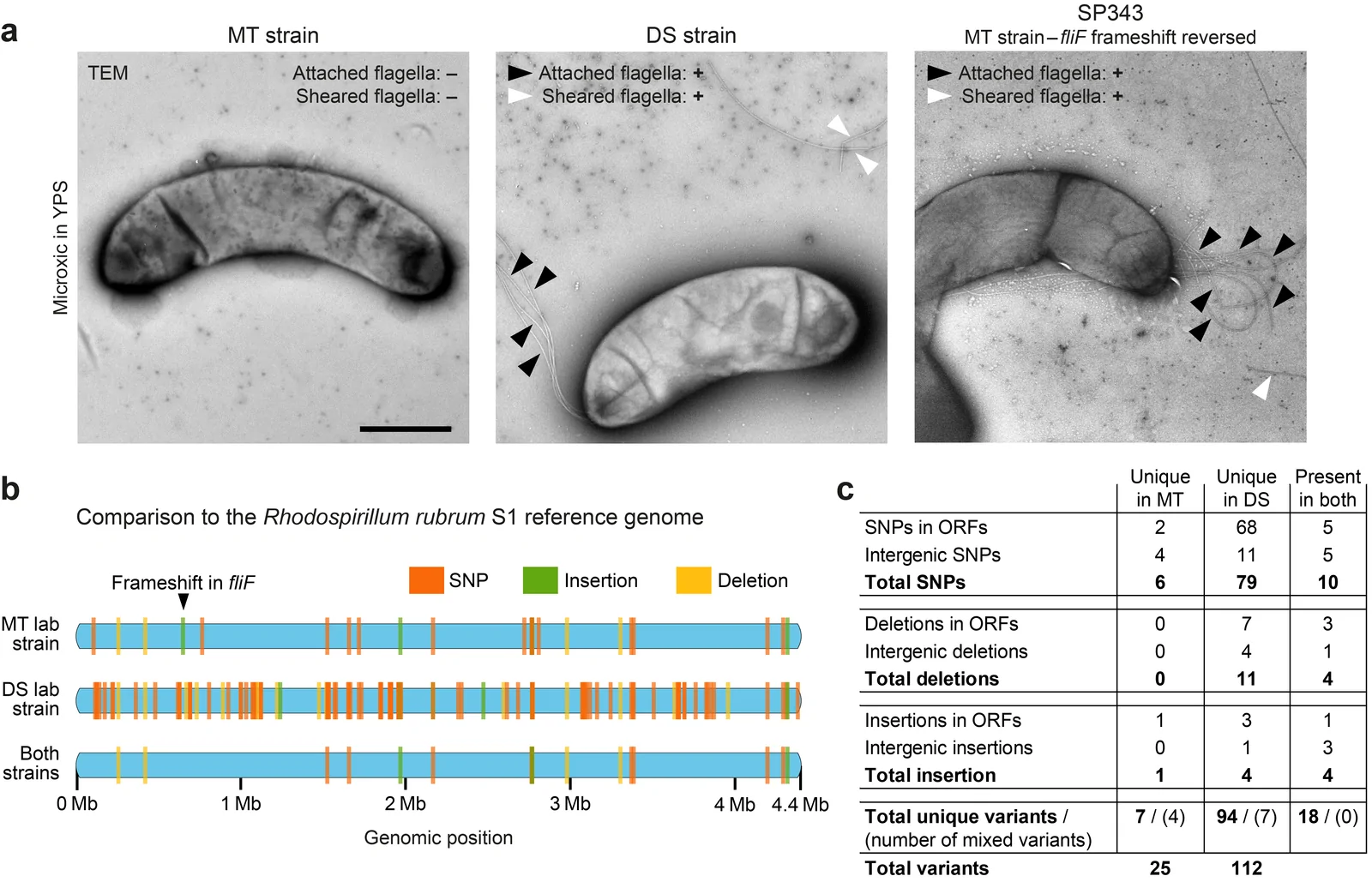



## Description

Culture collections and research laboratories typically subject bacterial strains to periodic cycles of revival and re-preservation to ensure long-term viability. However, serial passaging can introduce spontaneous mutations that are propagated through subsequent generations (Marks et al., 2010; Kram et al., 2017; Knöppel et al., 2018; Ratib et al., 2021). When such mutations confer a selective fitness advantage under laboratory conditions, genomic variants can rapidly dominate the population, leading to significant evol­utionary divergence from the progenitor strain. This genetic drift may com­pro­mise experimental repro­ducibility and invalidate comparisons against established reference genomes.

As a case in point, while studying cell morphogenesis in the spiral-shaped bacterium *Rhodospirillum rubrum* S1 (Pöhl et al., 2024) – the type strain of the species *Rhodospirillum* (Molisch, 1907; van Niel, 1944; Pfennig & Trüper, 1971) – we observed that a culture of this strain originally acquired from the Leibniz Insti­tute DSMZ (Germany) in 2016 under catalogue number DSM 467 lacked its previously reported fla­gella-medi­ated swim­ming motility (Lee & Fitzsimons, 1976). The cells were obtained in a lyo­philized state, revived in liquid medium, and immediately preserved in our labora­tory collection as a cryo­stock (hereafter designated as the MT strain) to minimize genetic diver­gence from the repository refer­ence, rendering this lineage effectively identical to DSM 467. Importantly, a culture newly procured from the DSMZ in 2026 displayed the same non-motile pheno­type, confirming that this defect did not arise spontaneously during laboratory handling or cryo­preser­vation. By con­­trast, normal swim­ming behav­ior was observed for a widely studied *R. rubrum* S1 labor­atory lineage provided by the research group of Dirk Schüler (Bayreuth, Ger­many) that was likely sourced from the American Type Culture Collec­tion in the mid-1960s (hereafter designated as the DS strain). In line with these obser­va­­tions, transmission elec­tron micros­copy revealed that the MT strain failed to assemble fla­gellar fila­ments, whereas the DS strain showed the characteristic mono­polar or bi­polar fla­gel­­lation pattern docu­mented in the literature ( **Figure 1a** ; Lee & Fitzsimons, 1976). To identify the genetic basis of this pheno­­typic differ­ence, we sequenced the genomes of both laboratory strains and compared them to the *R. rubrum* S1 reference genome (Munk et al., 2011), which is derived from an *R. rubrum* S1 stock deposited at the American Type Culture Collection (ATCC 11170).

Comparative sequence analysis revealed that both strains deviated from the reference genome at multi­ple loci ( **Figure 1b,c** ). A subset of these variations was strain-specific, whereas 18 mutations were shared, suggesting that genetic drift had already commenced within the culture collection prior to further diver­gence of the strains during independent maintenance in the respective laboratories. The shared devia­tions comprise 10 single nucleotide polymorphisms (SNPs). Five are located in intergenic regions, while the remaining five introduce non-synonymous mutations in predicted open reading frames (ORFs), result­ing in amino acid substi­tu­tions in the putative gene products (see **Extended Data** ). Both strains addi­tionally har­bor insertions and deletions that disrupt ORF *Rru_A0205* (DnaJ-like protein), fuse *Rru_A0341* (CRISPR-associated protein) with *Rru_A0342* (uncharacterized), ex­tend the reading frame of *Rru_A2352* (unchar­acterized) and fuse *Rru_A2834* (uncharacterized) with *Rru_A2835* (MotB-like protein).

Beyond the shared deviations, the genome of the MT strain exhibited seven unique differences relative to the reference sequence ( **Figure 1b,c** ). Six of these are SNPs located either in intergenic regions or within ORFs, where they result in amino acid substitutions. The remaining deviation was a single base-pair inser­tion within the gene for the flagellar MS-ring protein FliF (Homma et al., 1987; Ueno et al., 1992), which leads to a shift in the reading frame, thereby disrupting FliF function. The MS-ring forms the foun­dation of the bacterial flagellum, anchoring the motor in the inner membrane and serving as the base for the assembly of structural com­ponents such as the flagellar rod and filament (Francis et al., 1992; Kubori et al., 1992; Ueno et al., 1994). The disruption of *fliF* in the MT strain thus abolishes flagellar biogenesis, in agreement with the lack of flagella observed by electron microscopy ( **Figure 1a** ). Notably, flagellar assem­bly and motility were fully restored by reverting the frameshift mutation in *fliF* back to the reference genome sequence ( **Figure 1a** ), confirming the absence of other mutations deleterious to fla­gel­lar motility.

As expected, the DS strain did not show any mutations in motility-related genes, but it stood out due to its high frequency of unique deviations from the reference genome ( **Figure 1b,c** ). In total, the strain harbors 79 unique SNPs, with 11 located in intergenic regions and 68 within open reading frames (ORFs). While the majority of the latter result in silent or non-synonymous mutations, three have more severe functional consequences: two introduce nonsense mutations that truncate *Rru_A1282* (a VirG-like pro­tein) and *Rru_A1603* (a PhoH-like protein), while the third disrupts the start codon of *Rru_A2703* (a TrmH-like protein), thereby pre­cluding the formation of the gene product. In addition to SNPs, the DS strain harbors 3 unique deletions, which shor­ten ORFs *Rru_A0918* (histidine kinase) and *Rru_A3395* (LuxR-like transcriptional regulator) and intro­duce a frameshift in *Rru_A1277* (histidine kin­ase), completely dis­rupting its function. Furthermore, the strain exhibits 4 unique insertions. One is located in an intergenic region, while two others induce frame­shifts that disrupt *Rru_A1034* (a histidine kinase) and *Rru_A1843* (an OmpA/MotB-like protein). Finally, the DS strain is characterized by a 1,416 bp insertion within *Rru_A2108* (a histidine kinase). This segment exhibits high similarity (93% coverage; 73.56% identity; E-value 2E-125) to a genom­ic region in *Pararhodospirillum photometricum* DSM 122 (positions 1,859,748–1,861,060), suggesting that it was acquired via horizontal gene transfer.

Collectively, these findings illustrate the rapid pace of bacterial genome evolution under laboratory condi­tions. Common cultivation practices subject bacteria to various selective pressures, such as the demand for rapid planktonic growth in rich media under fixed environmental conditions, the need to withstand extended incubation in stationary phase on nutrient agar plates, and the requirement to survive pro­longed cooling and repeated freeze-thaw cycles during strain maintenance. Adaptive mutations can rapid­ly accumulate during these stress phases and become fixed in subsequent generations. Consistent with this notion, both laboratory strains accumulated mutations in transcriptional regulators and histidine kinases, which are critical for environmental stress sensing and adaptation. To mitigate the selection of genomic vari­ants, new strains should be cryopreserved at the earliest opportunity after their isolation, keeping the number of serial passages to a minimum. Moreover, practices such as the long-term storage of cultures in stationary phase at room temperature, which are still commonly encountered for photo­synthetic bac­teria, should be strictly avoided.

Our data demonstrate that, despite sharing the same origin, *R. rubrum* S1 laboratory and repository strains commonly used in research and biotechnology have diverged significantly over the past decades. The variations in their genomic sequences represent a critical factor that should be carefully considered when comparing experi­mental results obtained with strains from different lineages.

## Methods

**Growth conditions.** For the preparation of genomic DNA, the variants of *R. rubrum* S1 (DSM 467) (Molisch, 1907; van Niel, 1944; Pfennig & Trüper, 1971) investigated in this study were grown to exponential phase in the dark at 28 °C in liquid Tryptic Soy (TS) medium (1.7% tryptone, 0.3% soytone, 0.25% dextrose, 0.5% NaCl, 0.25% K _2_ HPO _4_ ) shaking at 210 rpm. To visualize flagella, cells were grown in a MIR-154 daylight-incubator (Panasonic, Japan) at 28 °C in liquid YPS medium (0.3% yeast extract, 0.3% peptone, 1 mM CaCl _2_ , 1 mM MgSO _4_ ) rotating at 20 rpm in Falcon tubes under microoxic conditions (14 ml culture in closed 15 ml Falcon tubes).

**Plasmid and strain construction.** The bacterial strains, plasmids and oligonucleotides used in this study are listed in the Reagents section. To construct plasmid pSP250, used to reverse the frameshift mutation in the *fliF* gene, the regions flanking base pair 642,095 of the *R. rubrum* S1 MT laboratory strain chromosome were PCR-amplified using pri­mer pairs oSP895/oSP896 and oSP897/oSP898. The amplification products, which carried a single-base-pair deletion that removed the insertion disrupting the *fliF* reading frame, was cloned into the *sacB* -containing allelic re­placement vector pNPTS138 (M.R.K. Alley, unpublished) via Gibson assembly, using HindIII- and NheI-digested vector DNA (Gibson et al., 2009) and *E. coli* TOP10 (Thermo Fisher Scientific, USA) as the cloning host. The resulting plasmid was verified by DNA sequencing. To construct the *fliF* revertant SP343, the MT strain was transformed with plasmid pSP250 via conjugation, employing the donor strain *E. coli* WM3064 (Pöhl et al., 2024). Single integrants were subjected to allelic replace­ment based on sucrose counterselection to select for a second recombination event (Cserti et al., 2017; Jung et al., 2015). Clones that had undergone double homologous recombination, thereby restoring the wild-type *fliF* allele, were identified by colony PCR, followed by sequencing of the PCR pro­ducts.

**Transmission electron microscopy.** Cells were grown under microoxic conditions at 28 °C in liquid YPS medium to an OD _600_ of 0.4, harvested by centrifugation for 3 min at 2500 xg, and then resuspended in the same, the 0.05-fold or the 0.0125-fold volume in fresh YPS medium, using plastic tips with cut-off tips to avoid the shearing of flagella. For imaging, 50 μl aliquots of the samples were transferred to parafilm. Subsequently, glow-discharged carbon-coated copper 300 mesh grids were placed upside-down on top of the sample (S162-3; Plano Wetzlar, Germany). After 10 min of incubation, the grids were washed twice with water and treated for 10 sec with 2% (w/v) uranyl acetate. Micrographs were taken with a JEM-1400 transmission electron microscope (JEOL, Japan) at an acceleration voltage of 100 kV. Fiji 1.54f (Schindelin et al., 2012) was used for data analysis.

**Genome sequencing and comparison.** Genomic DNA was purified with the NucleoSpin® Microbial DNA kit (Macherey-Nagel, Germany; Ref: 740235) according to the manufacturer’s instructions. Bacterial genome sequencing was performed by Plasmidsaurus (Louisville, KY, USA) integrating long-read Oxford Nanopore and short-read Illumina se­quencing technologies, followed by custom analysis and annotation (Supplementary Data). Clair3 (Zheng et al., 2022) was used to compare the gen­omes obtained to the *R. rubrum* S1 reference sequence (GenBank accession no. NC_007643.1; Munk et al., 2011) and identify SNPs and small indels.

**Availability of biological material.** The bacterial strains used in this study are available from the corresponding author upon request.

**Data availability.** The genome sequences of the two *R. rubrum* S1 laboratory strains analyzed in this study have been deposited to the European Nucleotide Archive (ENA) with the dataset identifier PRJEB114701 [ https://www.ebi.ac.uk/ena/browser/view/PRJEB114701 ]. All other data generated in this study are included in the manu­script or the **Extended Data** file.

## Reagents

**Strains**

**Table:** 

**Strain**	**History/genotype**	**Source**
*Rhodospirillum rubrum* S1 (DSM 467)	This strain is the neotype strain of *R. rubrum* provided by the Leibniz Institute DSMZ-German Collection of Microorganisms and Cell Cultures (DSMZ, Germany) under catalogue number DSM 467. DSMZ originally obtained the culture from the American Type Culture Collection (ex ATCC 11170). A comparison of its DSM catalogue number to nearby entries suggests an acquisition date between 1973 and 1974.	DSMZ
*Rhodospirillum rubrum* S1 (MT strain)	The strain was obtained from the DSMZ in 2017 as a lyophilized sample under catalogue number DSM 467. The cells were resuspended and revived in tryptic soy (TS) medium prior to direct storage as a master cryostock.	DSMZ
*Rhodospirillum rubrum* S1 (DS strain)	The DS strain was obtained in the early 2010s from Hartmut Grammel (Hochschule Biberach, Germany), who had received it from Robin Ghosh (University of Stuttgart, Germany) around the same time. Ghosh, who has published research on this strain since 1993, originally acquired it from Reinhard Bachofen (University of Zurich, Switzerland). Bachofen initiated his work on this specific lineage in 1964. Since the original culture was sourced prior to the establishment of the DSMZ, it probably tracks back to the original ATCC 11170 repository stock (personal communication with H. Grammel and R. Bachofen).	Dirk Schüler (University of Bayreuth, Germany)
*Rhodosprillum rubrum* SP343	Derivative of the *R. rubrum* S1 MT laboratory strain carrying the wild-type *fliF* allele	This study
*Escherichia coli* TOP10	F ^–^ *mcrA* Δ( *mrr* - *hsd* RMS- *mcr* BC) Φ80 *lac* ZΔM15 Δ *lac* X74 *recA* 1 *araD* 139 Δ( *ara-leu* ) 7697 *galU* *galK* *rpsL* (Str ^R^ ) *endA* 1 *nup* G	Thermo Fisher Scientific
*Escherichia coli* WM3064	*thrB1004 pro thi rpsL hsdS lacZ* ΔM15 RP4–1360 Δ( *araBAD* )567 Δ *dapA* 1341::[ *erm pir* (wt)]	W. Metcalf, unpublished

**Plasmids**

**Table:** 

**Designation**	**Description**	**Source**
pNPTS138	*sacB* ‐containing suicide vector used for double homologous recombination, pUC *ori* , Kan ^R^	M.R.K. Alley, unpublished
pSP250	pNPTS138 derivative used to restore the wild-type *fliF* allele in the MT strain by double homologous recombination	This study

**Primers**

**Table:** 

**Designation**	**Sequence** (5′ to 3′)	**Source**
oSP895	GCAATTGAAGCCGGCTGGCGCCAGAACTGCATGTTCACCACTTCGATC	This study
oSP896	CATCCAGGGGGTGGAGGGCGCCCGCG	This study
oSP897	GCGCCCTCCACCCCCTGGATGGTGCGCACG	This study
oSP898	CCGGAGACGCGTCACGGCCGAAGCCGCGAAGTCCAGAAGGGCG	This study

## Data Availability

Description: This spreadsheet details both the shared and unique variants identified across both strains, alongside quality control metrics for the underlying sequencing data. Additionally, it specifies the affected open reading frames (ORFs) and the predicted functional annotations of the encoded proteins (XLSX file; Microsoft Excel).. Resource Type: Dataset. DOI: https://doi.org/10.22002/mhmjd-4pr39
